# Multimodal evaluation of periosteal versus collagen membrane coverage in atelocollagen‐associated autologous chondrocyte implantation for large knee cartilage defects

**DOI:** 10.1002/jeo2.70924

**Published:** 2026-09-25

**Authors:** Takuma Kaibara, Eiji Kondo, Yuki Ogawa, Masatake Matsuoka, Koji Iwasaki, Tomohiro Onodera, Yoshitaka Oda, Zen‐ichi Tanei, Daisuke Momma, Shinya Tanaka, Kazunori Yasuda, Tomonori Yagi, Norimasa Iwasaki

**Affiliations:** ^1^ Department of Orthopaedic Surgery Hokkaido University Graduate School of Medicine Sapporo Hokkaido Japan; ^2^ Centre for Sports Medicine Hokkaido University Hospital Sapporo Hokkaido Japan; ^3^ Department of Functional Reconstruction for the Knee Joint Hokkaido University Sapporo Hokkaido Japan; ^4^ Department of Cancer Pathology, Faculty of Medicine Hokkaido University Sapporo Hokkaido Japan; ^5^ Knee Research Center Yagi Orthopaedic Hospital Sapporo Hokkaido Japan

**Keywords:** autologous chondrocyte implantation, cartilage defect, collagen membrane, histological evaluation, periosteum

## Abstract

**Purpose:**

Atelocollagen‐associated autologous chondrocyte implantation (A‐ACI) with periosteal coverage shows favourable outcomes for large knee cartilage defects but is associated with complications such as graft hypertrophy. Collagen membrane coverage remains under‐investigated in this technique. This study aimed to evaluate periosteal versus collagen membrane coverage using clinical, radiological, arthroscopic and histological assessments.

**Methods:**

Between 2014 and 2022, patients with ≥4.0 cm^2^ full‐thickness chondral lesions underwent A‐ACI using periosteal (*n* = 13) or collagen membrane (*n* = 13) coverage in a non‐randomized sequential comparative study. Clinical and radiological outcomes were assessed at 12 and 24 months using the Lysholm score, Knee injury and Osteoarthritis Outcome Score and Magnetic Resonance Observation of Cartilage Repair Tissue (MOCART) 2.0 score. Second‐look arthroscopy included International Cartilage Repair Society (ICRS) and Oswestry Arthroscopy Score (OAS) assessments, with histological analysis using the ICRS II scoring system. Minimum follow‐up was 24 months.

**Results:**

Both groups demonstrated significant clinical improvement at 24 months without significant between‐group differences. MOCART 2.0 scores were significantly higher in the collagen membrane group at 12 and 24 months (83 vs. 60 at 24 months, *p* = 0.001). OAS scores were significantly higher in the collagen membrane group (8.5 vs. 6.7, *p* = 0.034), while ICRS scores were comparable. Histological parameters, including tissue morphology, basal integration and subchondral bone quality, were significantly higher in the collagen membrane group. Complication profiles differed: graft hypertrophy and delamination in the periosteal group (46%) versus subchondral bone cysts in the collagen membrane group (15%). Collagen membrane coverage remained associated with higher MOCART 2.0 scores after limited adjustment for concomitant osteotomy.

**Conclusions:**

The primary clinical outcomes improved similarly in both groups at two years without meaningful between‐group superiority. While collagen membrane coverage was associated with higher MOCART 2.0 scores after adjustment for concomitant osteotomy, all structural evaluations should be regarded strictly as secondary and exploratory.

**Level of Evidence:**

Level III.

**Abbreviations**
ACL: anterior cruciate ligament
ADLs: activities of daily living
AOT: autologous osteochondral transplantation
A‐ACI: atelocollagen‐associated autologous chondrocyte implantation
BMI: body mass index
HTO: high tibial osteotomy
ICRS: International Cartilage Repair Society
KOOS: Knee injury and Osteoarthritis Outcome Score
LFC: lateral femoral condyle
MFC: medial femoral condyle
MOCART 2.0: Magnetic Resonance Observation of Cartilage Repair Tissue 2.0
MRI: magnetic resonance imaging
OAS: Oswestry Arthroscopy Score
OCD: osteochondritis dissecans
PCL: posterior cruciate ligament
QOL: quality of life

## INTRODUCTION

Autologous chondrocyte implantation (ACI) has evolved through several generations for the treatment of large cartilage defects [8, 15, 28, 33]. First‐generation ACI using periosteal coverage was associated with complications such as graft hypertrophy in up to 40% of cases [14, 22, 37, 56], leading to the development of second‐generation techniques utilizing collagen membrane coverage, which reduced complication rates [14, 22, 56]. Although favourable long‐term outcomes have been reported, ACI has yet to consistently regenerate cartilage with native hyaline quality, highlighting the need for further optimization [20, 54].

In pursuit of improved cartilage regeneration, atelocollagen‐associated ACI (A‐ACI) was developed, characterized by three‐dimensional ex vivo culture of harvested chondrocytes in atelocollagen prior to implantation [39, 40]. Although periosteal‐covered A‐ACI has shown favourable clinical outcomes in multiple studies, complications such as graft hypertrophy, which has been reported in 11%–30% of cases, remain a significant concern, potentially attributable to the use of periosteum [1, 17, 18, 50, 51]. Furthermore, histological evaluations have revealed that the regenerated tissue after A‐ACI does not fully replicate the zonal architecture or matrix composition of native hyaline cartilage [1, 17].

To address these potentially periosteal coverage‐related issues in A‐ACI, porcine‐derived Type I/III collagen membranes (Chondro‐Gide, Geistlich Biomaterials) were approved as an alternative by Japan National Health Insurance in 2019. A collagen membrane‐covered conventional ACI using a cell suspension has demonstrated reduced complication rates compared to periosteal coverage [12, 13]. Furthermore, recent studies suggest additional benefits including superior long‐term outcomes at 10 years [36] and better histological quality including cellular morphology and hyaline cartilage formation [31] compared to periosteal coverage. These findings suggest that collagen membrane coverage may offer similar advantages in advanced A‐ACI techniques. However, the optimal coverage material may differ when applied to three‐dimensionally cultured gel‐form implants, given the differences in graft properties and the biological interaction between the membrane and the underlying graft compared to conventional cell‐suspension ACI. Only one study to date has evaluated collagen membrane coverage in A‐ACI using three‐dimensionally cultured chondrocytes [18]. While that study reported comparable clinical outcomes and superior arthroscopic scores with collagen membrane coverage, magnetic resonance imaging (MRI) and histological assessment were not performed, leaving the quality of regenerated cartilage largely unknown.

The purpose of this study was to perform a multimodal post‐operative assessment of periosteal‐ versus collagen membrane‐covered A‐ACI using clinical outcomes, MRI, arthroscopy and histological evaluation, to clarify the impact of membrane choice on the quality of regenerated cartilage. We hypothesized that collagen membrane‐covered A‐ACI would yield clinical outcomes comparable to periosteal‐covered A‐ACI, but with superior cartilage repair quality and a lower risk of complications.

## METHODS

### Study design

This prospective, comparative cohort study included patients who underwent A‐ACI performed by a single consultant orthopaedic surgeon (E.K.) between March 2014 and May 2022. Patients were eligible if they presented with full‐thickness chondral lesions in the knee with a preoperative MRI‐measured area of 4.0 cm^2^ or greater, resulting from trauma or osteochondritis dissecans (OCD). The actual lesion size was subsequently verified and documented via direct measurement during surgery. Patients with cartilage lesions due to osteoarthritis, rheumatoid arthritis or other systemic joint disorders were excluded. Concomitant procedures were performed when clinically indicated to optimize the biomechanical environment for cartilage repair. Preoperatively, all patients underwent standing anteroposterior and lateral radiographs of the knee, as well as full‐length weight‐bearing lower extremity radiographs. Coronal alignment and the required correction angle were evaluated according to the principles of bone deformity correction [42]. When mechanical unloading of the chondral lesion was deemed necessary, a realignment osteotomy tailored to the primary site of deformity in the femur or tibia was performed to shift the mechanical axis to 62%–65%. High tibial osteotomy or distal femoral osteotomy was selected based on the specific deformity analysis. High tibial osteotomy was performed using either an opening‐wedge or inverted V‐shaped technique depending on the required correction angle and the presence of patellofemoral osteoarthritis [21]. Furthermore, for large patellofemoral cartilage defects without patellofemoral maltracking, an anteriorization of the tibial tubercle using the modified Maquet procedure [29] was considered to mechanically unload the patellofemoral joint according to previously reported indications [55]. Additionally, concurrent ligament reconstruction or meniscal surgery was performed for patients with knee instability or meniscal pathology, respectively.

The study consisted of two consecutive treatment series: between March 2014 and January 2019, chondrocyte implants were covered by autologous periosteum (Group P), while from February 2019 to May 2022, following its approval by Japan National Health Insurance, a porcine‐derived collagen membrane (Group C) was used. Post‐operatively, patients were evaluated in the outpatient clinic at 3, 6, 12 and 24 months, and annually thereafter when feasible. Patient‐reported clinical outcomes, collected via paper‐based questionnaires at these visits, were defined as the primary endpoints. To assess the structural quality of the repair tissue, radiological, arthroscopic and histological evaluations were defined collectively as secondary, exploratory endpoints. Specifically, post‐operative MRI was recommended and performed conditional on patient consent. Second‐look arthroscopy and histological evaluation were performed in patients requiring additional procedures or hardware removal. All complications were prospectively documented throughout the follow‐up period. In this study, clinical failure was defined as the requirement for additional surgical intervention due to graft‐related complications. The study protocol was approved by the institutional review board, and written informed consent was obtained from all participants.

### Surgical procedure

**Figure 1:**
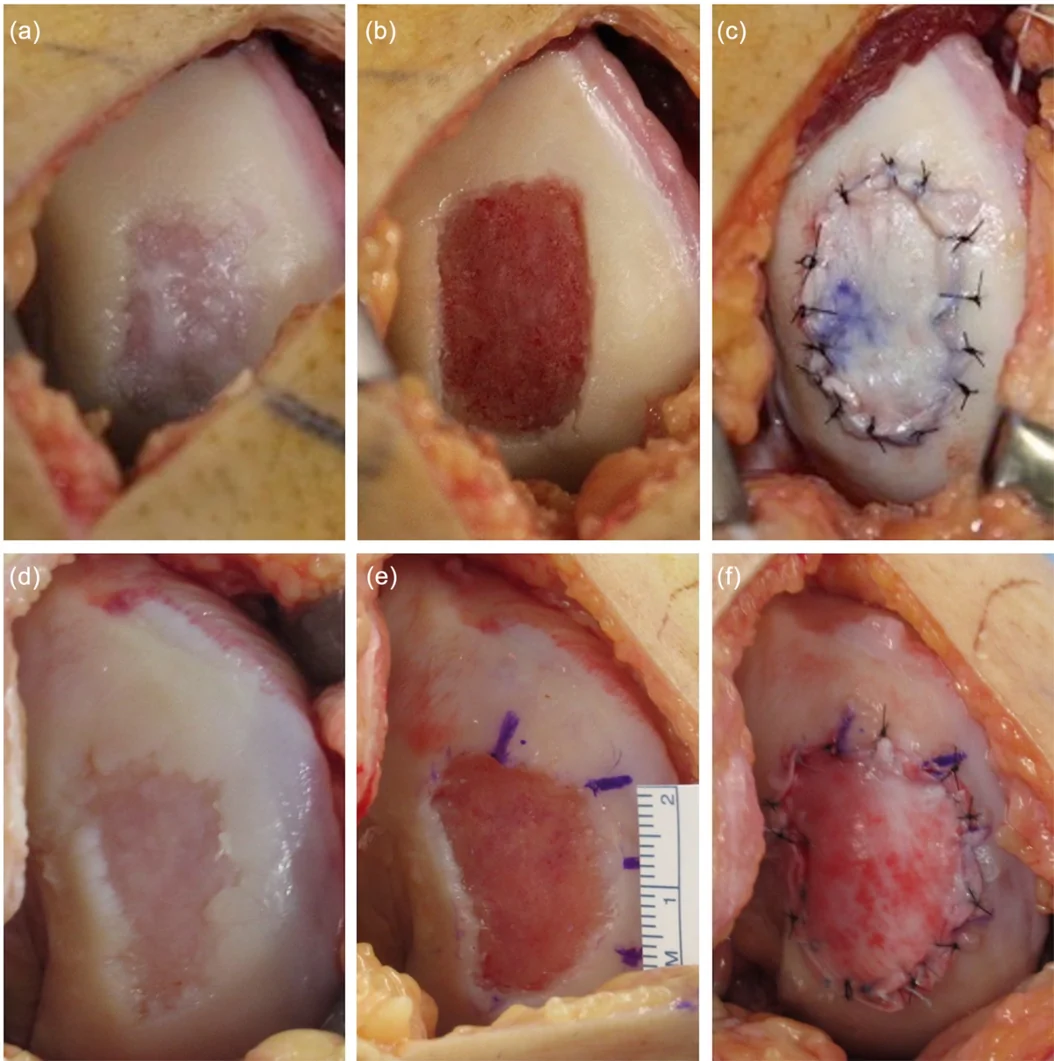
Intraoperative images of atelocollagen‐associated autologous chondrocyte implantation using periosteal (a–c) and collagen membrane (d–f) coverage. Cartilage lesions were exposed via minimal arthrotomy (a, d), debrided to healthy surrounding cartilage and subchondral bone (b, e), and subsequently covered with either periosteum (c) or collagen membrane (f) following implantation of cultured chondrocytes embedded in atelocollagen gel.

The surgical procedure consisted of two stages following a previously described protocol [17]. Briefly, the first stage was to harvest more than 0.4 g of articular cartilage from a non‐weight‐bearing region of the femoral condyle, which was delivered to a designated facility (Japan Tissue Engineering Co., Ltd.). Chondrocytes were isolated and cultured three‐dimensionally with atelocollagen solution (3% type I collagen; Koken) for 4 weeks, resulting in disk‐shaped implants (diameter: 25 mm, thickness: 1.4–2.4 mm). In the second stage, through a minimal arthrotomy, the cartilage defect was exposed (Figure 1a,d) and debrided to healthy surrounding cartilage and subchondral bone (Figure 1b,e). The cultured chondrocytes embedded in atelocollagen gels were then implanted into the defect and covered with either periosteum (Group P, Figure 1c) or a commercially available collagen membrane (Chondro‐Gide, Geistlich Biomaterials; average thickness: 0.5 mm) (Group C, Figure 1f). For Group P, the periosteum was harvested from the anteromedial surface of the tibia and shaped to match the defect contour. Both types of covering membranes were secured using the same combined technique. The membrane was first secured on one side to the surrounding cartilage rim using transosseous pull‐out sutures and, when necessary, supplemented with 1.4‐mm diameter suture anchors (JuggerKnot® Soft Anchor System, Zimmer Biomet). After the atelocollagen gel implants were inserted into the defect, the remaining side of the membrane was sutured in the same manner to fully enclose the graft (Figure 1c,f). In both groups, the final implant surface was positioned level with the surrounding articular cartilage.

### Post‐operative rehabilitation protocol

Post‐operative rehabilitation was performed according to a standardized protocol, with modifications based on any concomitant procedures. Range of motion exercises were initiated 1 week after surgery. Partial weight bearing was allowed at 4 weeks, progressing to full weight bearing at 6 weeks post‐operatively. Return to sporting activities was allowed at 12 months post‐operatively, provided that both clinical and radiological outcomes were acceptable. When concomitant procedures were performed, the more restrictive timeline among the combined procedures was followed for each rehabilitation component. For weight bearing, the A‐ACI protocol was consistently the most conservative and therefore determined the timeline regardless of concomitant procedures. For range of motion, modifications were dictated by the concomitant procedure: in patients with concomitant meniscal repair, range of motion was restricted until 3 weeks and then limited to 0–90°, progressing to unrestricted range of motion at 12 weeks; in patients with concomitant ACL reconstruction, range of motion was limited to 0–30° initially, progressing to full range of motion at 12 weeks; in patients with concomitant high tibial osteotomy without meniscal repair, no additional range of motion restrictions were applied.

### Clinical evaluation

Clinical outcomes were evaluated using the Lysholm score and the Knee injury and Osteoarthritis Outcome Score (KOOS). The KOOS consists of five subscales: Symptoms, Pain, Function in Daily Living (ADL), Function in Sport and Recreation (Sport/Rec) and knee‐related Quality of Life (QOL).

### Radiological evaluation

The MRI of the knee was performed using a dedicated knee coil to assess the repaired cartilage tissue. The MRI protocol included T1‐weighted, T2‐weighted and fat‐suppressed proton density sequences. Slice thickness was set at 3.0 mm with an interslice gap of 0.3 mm. The quality of the repair tissue was evaluated using the Magnetic Resonance Observation of Cartilage Repair Tissue 2.0 (MOCART 2.0) scoring system [47] at 12 and 24 months post‐operatively.

### Second‐look arthroscopic evaluation

Second‐look arthroscopy was performed in cases requiring additional procedures or hardware removal. Arthroscopic findings were evaluated by the operating surgeon (E.K.) using the Oswestry Arthroscopy Score (OAS) and the International Cartilage Repair Society (ICRS) scoring system [48, 53]. The OAS assesses five parameters, including graft hypertrophy, with a maximum score of 10 points, whereas the ICRS score does not include graft hypertrophy assessment. The ICRS score is based on a 12‐point scale, categorized into four grades: Grade I (12 points, normal), Grade II (8–11 points, nearly normal), Grade III (4–7 points, abnormal) and Grade IV (1–3 points, severely abnormal).

### Histological evaluation

Needle biopsy specimens were obtained from the centre of the implanted grafts during second‐look arthroscopy. Not all patients who underwent second‐look arthroscopy had biopsy specimens obtained, as tissue sampling was performed only in patients who provided additional informed consent for the procedure. Longitudinal sections were stained with hematoxylin and eosin (H&E) and safranin‐O to evaluate tissue architecture and proteoglycan content, respectively. Histological assessment was performed using the ICRS II scoring system, which employs a 0–100 visual analogue scale for each of 14 parameters [27]. Two experienced pathologists (Y.O. and Z.T.), blinded to patient information, independently evaluated the sections and subsequently reached consensus on the ICRS II scores through discussion. Although all specimens underwent identical staining protocols, histological processing was performed at different time points throughout the study period, and minor variations in staining conditions between batches cannot be entirely excluded.

### Statistical analysis

Statistical analyses were performed using IBM SPSS Statistics version 26 (IBM Corp.) and R version 4.5.1 (R Foundation for Statistical Computing). Data normality was assessed using the Shapiro‐Wilk test. For normally distributed continuous variables, between‐group comparisons were performed using the independent *t* test; for non‐normally distributed data, the Mann–Whitney *U* test was used. Categorical variables were compared using Fisher's exact test or chi‐square test as appropriate. Longitudinal changes within each group were evaluated using the Friedman test, followed by post hoc Wilcoxon signed‐rank tests with Bonferroni correction for multiple comparisons. A post hoc power analysis was performed using the Lysholm score as the primary outcome, with a clinically meaningful between‐group difference of 10.5 points based on the minimal clinically important difference [41] and a standard deviation of 9.5 points based on post‐operative scores derived from prior A‐ACI literature [51], at a significance level of 0.05. Complication‐free survival was estimated using the Kaplan–Meier method, and differences between groups were assessed using the log‐rank test, performed using the survival and survminer packages in R. To evaluate the potential confounding effect of concomitant osteotomy, two additional analyses were performed. First, a sensitivity analysis was conducted by excluding all patients who underwent concomitant osteotomy and repeating all between‐group comparisons. Second, multivariable linear regression analyses were performed with each outcome score as the dependent variable and membrane type (collagen membrane vs. periosteum) and concomitant osteotomy (yes vs. no) as explanatory variables, to evaluate whether the association between membrane type and each outcome persisted after adjustment for osteotomy. No formal adjustment for multiple comparisons was applied to the secondary structural endpoints because these analyses were exploratory and the individual parameters were intended to assess distinct aspects of cartilage repair quality. Therefore, the corresponding *p* values should be interpreted as descriptive and hypothesis‐generating rather than confirmatory. Statistical significance was set at *p* < 0.05.

## RESULTS

### Patient demographics

**Figure 2:**
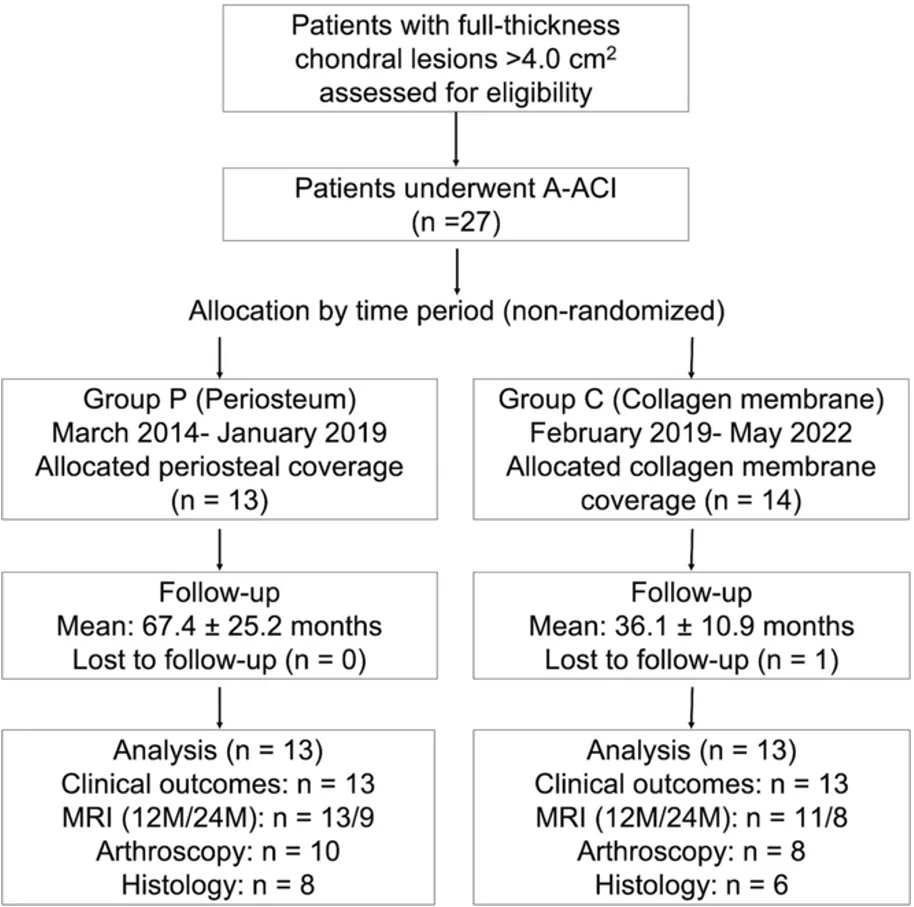
Participant selection and allocation flowchart. A‐ACI, atelocollagen‐associated autologous chondrocyte implantation; MRI, magnetic resonance imaging.

A total of 27 patients underwent A‐ACI with either periosteal (Group P, *n* = 13) or collagen membrane (Group C, *n* = 14) coverage (Figure 2). One patient in Group C was lost to follow‐up before the 12‐month evaluation and was excluded from the analysis. Group P included 11 men and 2 women with a mean age of 33.4 ± 12.3 years (range, 15–52), while Group C included 6 men and 7 women with a mean age of 43.8 ± 11.5 years (range, 15–59) (Table 1). No significant differences were observed between the groups in terms of age, sex distribution, body mass index, aetiology of the lesion, lesion location or defect size. The mean follow‐up duration was significantly longer in Group P (67.4 ± 25.2 months; range, 24–103) than in Group C (36.1 ± 10.9 months; range, 24–53) (*p* < 0.001). Concomitant procedures included osteotomy in 9 cases (Group P: 1, Group C: 8; *p* = 0.015). In Group C, three patients underwent simultaneous high tibial osteotomy and tibial tubercle osteotomy. Other concurrent procedures included autologous osteochondral transplantation and meniscal surgery in five cases each, anterior cruciate ligament reconstruction in three cases, and single cases of posterior cruciate ligament reconstruction and bone marrow stimulation. The post hoc power analysis indicated that 14 patients per group would be required to achieve 80% power. The present study with 13 patients per group achieved an estimated power of 77.2%. Detailed patient‐level data, including demographics, lesion characteristics, concomitant procedures, outcome scores and complications, were provided in Table S1.

**Table 1:** Demographic and clinical characteristics of patients.

	Group P (*n* = 13)	Group C (*n* = 13)	*p*
Age, years	33.4 ± 12.3	43.8 ± 11.5	0.062
Sex (male:female)	11:2	6:7	0.097
BMI, kg/m^2^	23.3 ± 4.3	25.6 ± 4.2	0.184
Cause, *n*			0.593
Trauma	10	12	
OCD	3	1	
Lesion site, *n*			0.189
MFC	5	5	
LFC	5	1	
Trochlea	5	8	
Defect size, cm^2^	5.6 ± 2.1	5.0 ± 1.2	0.334
Concomitant procedures, *n*			
Osteotomy	1	8	0.015
High tibial osteotomy	1	6	
Distal femoral osteotomy	0	1	
Tibial tubercle osteotomy	0	4	
AOT	3	2	1.000
Meniscal surgery	3	2	1.000
ACL reconstruction	2	1	1.000
PCL reconstruction	0	1	1.000
Bone marrow stimulation	1	0	1.000
F/U period, months	67.4 ± 25.2	36.1 ± 10.9	<0.001

*Note*: Values are expressed as mean ± standard deviation. Some patients had lesions at more than one location.

Abbreviations: A‐ACI, atelocollagen‐associated autologous chondrocyte implantation; ACL, anterior cruciate ligament; AOT, autologous osteochondral transplantation; BMI, body mass index; F/U, follow‐up; LFC, lateral femoral condyle; MFC, medial femoral condyle; OCD, osteochondritis dissecans; PCL, posterior cruciate ligament. Group C, collagen membrane‐covered A‐ACI; Group P, periosteal‐covered A‐ACI.

### Clinical evaluation

The median Lysholm scores improved from 74 to 81 and 93 points in Group P, and from 66 to 90 and 94 points in Group C at 12 and 24 months post‐operatively, respectively (Table 2). Group P showed significant improvement at 24 months post‐operatively (*p* = 0.043), while Group C improved at both 12 months (*p* = 0.042) and 24 months post‐operatively (*p* = 0.004) (Table 3). No significant differences were observed between groups at either time point. Regarding KOOS evaluation, all subscales showed significant improvement from baseline in both groups at 24 months post‐operatively (*p* ≤ 0.003 in Group P, *p* ≤ 0.033 in Group C). Pain, ADL and QOL in Group P showed significant improvement as early as 12 months post‐operatively. There were no significant differences between groups in any subscales at any time point. The between‐group differences in all clinical outcome measures at both time points did not exceed the minimal clinically important difference thresholds [41], supported by small effect sizes for all comparisons at 24 months (*r* ≤ 0.34).

**Table 2:** Clinical outcomes at baseline and follow‐up.

Outcome	Time point	Group P (*n* = 13)	Group C (*n* = 13)	Median Diff. (95% CI)	Effect size (*r*)	*p*
Lysholm	Preoperative	74 (72–79)	66 (50–68)	12.00 (0.00–23.00)	0.40	0.039
	12 Months	81 (74–95)	90 (88–90)	−5.00 (−15.00 to 9.00)	0.07	0.724
	24 Months	93 (80–99)	94 (83–95)	0.00 (−12.00 to 8.00)	0.01	0.927
KOOS						
Symptoms	Preoperative	68 (54–79)	54 (36–71)	10.70 (−7.10 to 25.03)	0.21	0.287
	12 Months	75 (64–86)	89 (79–89)	−10.70 (−21.40 to 3.50)	0.36	0.096
	24 Months	82 (75–96)	89 (80–95)	0.00 (−14.29 to 10.71)	0.01	0.955
Pain	Preoperative	78 (64–83)	64 (42–78)	13.89 (−0.00 to 27.74)	0.37	0.050
	12 Months	89 (69–97)	85 (81–90)	−0.00 (−16.67 to 11.11)	0.00	0.976
	24 Months	94 (86–97)	88 (83–95)	2.78 (−8.33 to 11.11)	0.15	0.538
ADL	Preoperative	79 (71–88)	72 (62–85)	7.34 (−5.91 to 19.09)	0.20	0.311
	12 Months	95 (85–99)	88 (85–96)	4.41 (−2.94 to 11.76)	0.25	0.235
	24 Months	97 (88–100)	93 (85–95)	3.93 (−1.47 to 11.74)	0.30	0.137
Sports/Rec	Preoperative	15 (10–50)	30 (15–55)	−5.00 (−25.00 to 10.00)	0.10	0.614
	12 Months	65 (30–80)	55 (30–70)	5.00 (−25.00 to 35.00)	0.10	0.647
	24 Months	75 (70–95)	60 (50–75)	20.00 (−0.00 to 30.00)	0.34	0.081
QOL	Preoperative	31 (25–38)	19 (13–31)	12.50 (−0.00 to 18.80)	0.37	0.057
	12 Months	56 (50–88)	50 (44–63)	12.50 (−6.20 to 31.30)	0.29	0.186
	24 Months	75 (63–94)	69 (56–75)	12.50 (−6.25 to 25.00)	0.27	0.186

*Note*: Values are expressed as median (interquartile range). Median Diff. (95% CI) represents the median difference between the groups with its 95% confidence interval. Effect size (*r*) was calculated from the Mann–Whitney *U* test.

Abbreviations: A‐ACI, atelocollagen‐associated autologous chondrocyte implantation; ADL, activities of daily living; Group C, collagen membrane‐covered A‐ACI; Group P, periosteal‐covered A‐ACI; KOOS, Knee Injury and Osteoarthritis Outcome Score; QOL, Quality of Life; Sports/Rec, Sports and Recreation.

**Table 3:** Longitudinal changes in clinical outcomes within each group.

			Post hoc analysis		
Group	Outcomes	Friedman test	Pre vs. 12 months	Pre vs. 24 months	12 vs. 24 months
Group P (*n* = 13)	Lysholm	0.041	0.287	0.043	1.000
	KOOS				
	Symptoms	0.002	0.509	0.003	0.150
	Pain	<0.001	0.024	<0.001	0.718
	ADL	<0.001	0.003	0.001	1.000
	Sports/Rec	0.001	0.150	0.001	0.287
	QOL	<0.0001	0.004	<0.001	1.000
Group C (*n* = 13)	Lysholm	0.002	0.042	0.004	1.000
	KOOS				
	Symptoms	0.018	0.069	0.033	1.000
	Pain	0.010	0.102	0.014	1.000
	ADL	0.008	0.053	0.012	1.000
	Sports/Rec	0.001	0.102	0.001	0.472
	QOL	0.013	0.135	0.015	1.000

*Note*: Values represent *p* values. Friedman test was used to assess overall changes over time, followed by post hoc pairwise comparisons with Bonferroni correction.

Abbreviations: A‐ACI, atelocollagen‐associated autologous chondrocyte implantation; ADL, activities of daily living; Group C, collagen membrane‐covered A‐ACI; Group P, periosteal‐covered A‐ACI; KOOS, Knee Injury and Osteoarthritis Outcome Score; QOL, Quality of Life; Sports/Rec, Sports and Recreation.

### Radiological evaluation

MRI evaluation was performed in 13 and 11 patients in Groups P and C at 12 months post‐operatively, and in 9 and 8 patients at 24 months post‐operatively, respectively. The median (interquartile range) MOCART 2.0 scores in Group P were 60 (55–65) at 12 months and 60 (55–70) at 24 months, while Group C achieved 70 (68–75) at 12 months and 83 (79–86) at 24 months post‐operatively. Group C demonstrated significant progressive improvement from 12 to 24 months post‐operatively (*p* = 0.025), while Group P showed no significant change over the same period (*p* = 0.739). Between‐group comparisons revealed significantly higher scores in Group C at both 12 months (*p* = 0.013) and 24 months (*p* = 0.001). Group C showed significantly higher scores in the bony defect and subchondral change subscales.

### Arthroscopic evaluation

Second‐look arthroscopy was performed in 10 out of 13 patients in Group P and 8 out of 13 patients in Group C at a mean of 28 ± 19 and 26 ± 16 months post‐operatively, respectively. In Group P, indications for second‐look arthroscopy included graft hypertrophy in three knees, graft delamination in three knees, hardware removal in three knees and loose body removal in one knee. In Group C, indications included subchondral bone cyst requiring additional intervention in two knees and hardware removal in six knees. The mean ICRS scores (mean ± SD) were 9.3 ± 3.4 in Group P and 11.3 ± 0.7 in Group C, showing no significant difference (*p* = 0.203). The ICRS grade distribution showed three Grade I (30%), five Grade II (50%) and one each of Grade III and Grade IV (10% each) in Group P, while Group C had four cases each of Grade I (50%) and Grade II (50%), with no Grade III or IV cases. The mean OAS scores (mean ± SD) were significantly higher in Group C than in Group P at 8.5 ± 1.1 and 6.7 ± 2.1, respectively (*p* = 0.034).

### Histological evaluation

**Figure 3:**
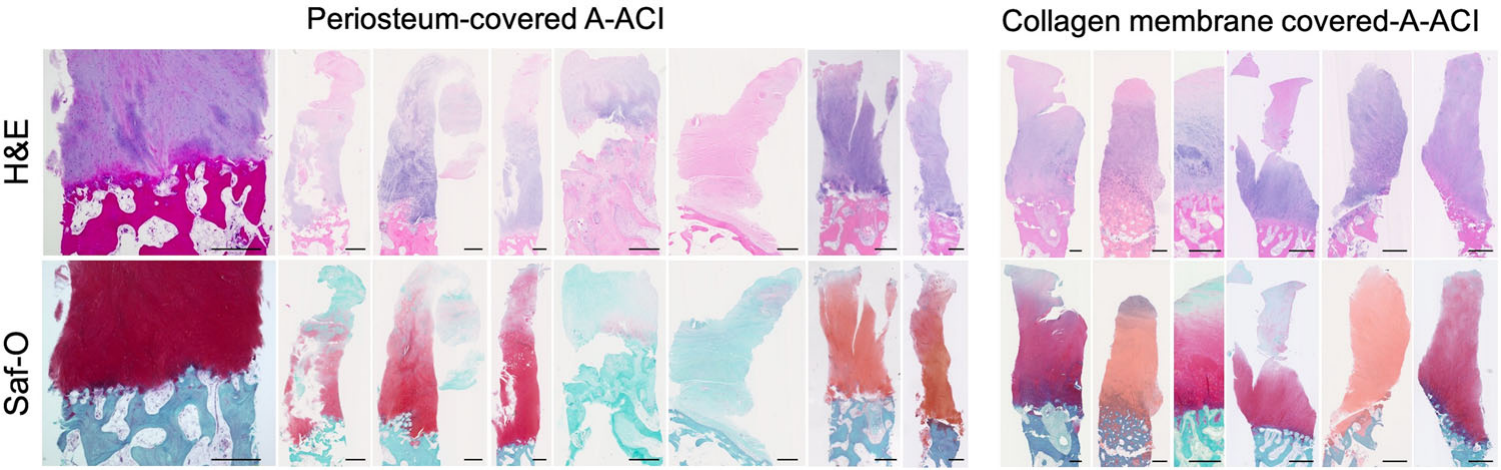
Histological comparison between periosteal‐covered A‐ACI (left, *n* = 8, mean 32 ± 18 months) and collagen membrane‐covered A‐ACI (right, *n* = 6, mean 26 ± 17 months) stained with hematoxylin and eosin (H&E, upper row) and Safranin‐O (Saf‐O, lower row). Safranin‐O‐positive cartilage‐like tissue was identified in six out of eight periosteal specimens and all collagen membrane specimens. Two periosteal specimens showed Safranin‐O‐negative fibrous repair tissue with minimal proteoglycan content. Note that magnification varies between specimens due to differences in biopsy size; scale bars were provided in each image for reference. Scale bar = 500 μm. A‐ACI, atelocollagen‐associated autologous chondrocyte implantation.

Tissue specimens were obtained from eight patients in Group P and six patients in Group C at a mean of 32 ± 18 and 26 ± 17 months post‐operatively, respectively. Safranin‐O‐positive cartilage‐like tissue was identified in six out of eight specimens in Group P and in all specimens in Group C (Figure 3). The typical organized zonal architecture observed in normal native cartilage was absent in both groups. One specimen in Group P showed fibrous tissue with vascularization, and three showed subchondral sclerosis. Safranin‐O positivity extending into the subchondral bone region was observed in some specimens of both groups. The median overall ICRS II assessment scores were similar between groups, while Group C showed significantly higher scores in tissue morphology, basal integration, subchondral bone abnormality and superficial assessment (*p* < 0.05) (Table 4).

**Table 4:** International Cartilage Repair Society (ICRS) II score.

	ICRS II score median (range)		
ICRS II parameter	Periosteum	Collagen	*p*
Tissue morphology	47.5 (5–90)	80 (70–100)	0.013
Matrix staining	65 (5–90)	80 (70–90)	0.108
Cell morphology	75 (5–100)	80 (60–85)	1.000
Chondrocyte clustering	97.5 (90–100)	100 (100)	0.142
Architecture of surface	92.5 (50–100)	90 (80–100)	0.755
Basal integration	80 (0–100)	100 (100)	0.020
Calcification front/tidemark	45 (0–60)	70 (30–70)	0.228
Subchondral bone abnormalities	87.5 (0–100)	100 (80–100)	0.029
Abnormal calcification	100 (100)	100 (100)	1.000
Inflammation	100 (100)	100 (100)	0.662
Vascularization in repair tissue	100 (5–100)	100 (100)	0.950
Surface/superficial assessment	30 (5–90)	70 (70–100)	0.013
Mid/deep zone assessment	55 (0–90)	80 (60–90)	0.059
Overall assessment	75 (1–90)	77.5 (70–90)	0.573

### Complications

**Figure 4:**
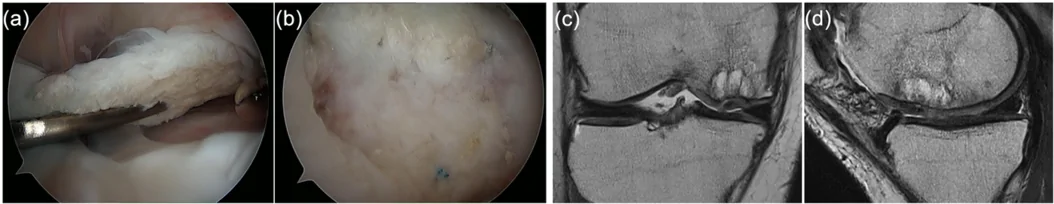
Complications observed in each group. (a, b) Graft delamination in Group P (periosteal coverage) involving a trochlear lesion, identified at 55 months post‐operatively. (a) The delaminated cartilage fragment was identified as a loose body in the suprapatellar pouch. (b) The implantation site exhibited a defect corresponding to the detached area. (c, d) Subchondral bone cyst formation in Group C (collagen membrane coverage), identified at 44 months post‐operatively. (c) Coronal and (d) sagittal T2‐weighted MRI images reveal a subchondral bone cyst measuring 10 × 16 × 18 mm in the medial femoral condyle. MRI, magnetic resonance imaging.

**Figure 5:**
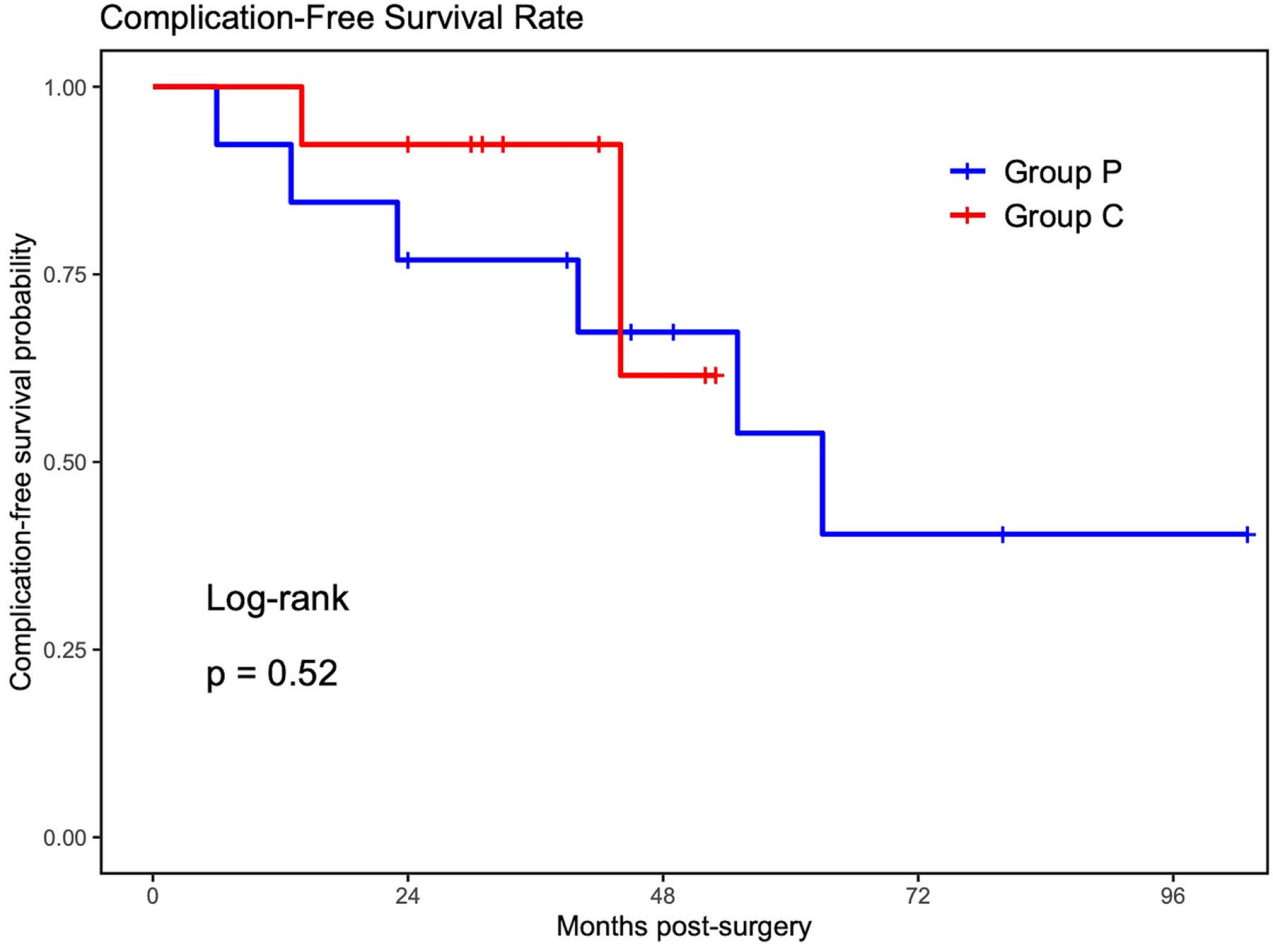
Kaplan–Meier curves for complication‐free survival in periosteal‐covered A‐ACI (Group P) and collagen membrane‐covered A‐ACI (Group C). A‐ACI, atelocollagen‐associated autologous chondrocyte implantation.

Although the difference did not reach statistical significance (*p* = 0.202), the complication rate tended to be higher in Group P than in Group C (46% vs. 15%), with an odds ratio of 4.71 (95% CI: 0.74–30.20). In Group P, three cases developed graft hypertrophy and three cases showed graft delamination requiring arthroscopic debridement (Figure 4A,B). In Group C, two cases developed progressively enlarging subchondral bone cysts at 14 and 44 months post‐operatively (Figure 4C,D). The cysts progressed to sizes of 11 × 13 × 13 and 10 × 16 × 18 mm, respectively, both requiring autologous osteochondral transplantation. To account for the significant difference in follow‐up durations, a Kaplan–Meier analysis was performed, demonstrating no significant difference in complication‐free survival between the groups over the entire follow‐up period (log‐rank test, *p* = 0.523) (Figure 5).

### Sensitivity and multivariable regression analyses

To evaluate the potential confounding effect of concomitant osteotomy, a sensitivity analysis was performed by excluding all patients who underwent concomitant osteotomy (Group P: *n* = 12, Group C: *n* = 5) (Table S2). No significant between‐group differences were observed in Lysholm scores at 12 or 24 months post‐operatively. Among the KOOS subscales, ADL and Sport/Recreation at 24 months were significantly higher in Group P than in Group C (*p* = 0.038 for both); however, preoperative scores in the remaining Group C patients were also significantly lower than those in Group P (Lysholm: *p* = 0.017; KOOS QOL: *p* = 0.016), and no significant differences were observed when the change from baseline to 24 months was compared (KOOS ADL: *p* = 0.562; Sport/Recreation: *p* = 0.368). The collagen membrane group maintained significantly higher MOCART 2.0 scores at both 12 months (median 70 vs. 60, *p* = 0.033) and 24 months (median 80 vs. 60, *p* = 0.020). Arthroscopic scores showed no significant differences between groups. For histological parameters, only 2 patients remained in Group C after osteotomy exclusion, precluding meaningful statistical comparison.

Multivariable linear regression analysis demonstrated an association between collagen membrane coverage and higher MOCART 2.0 scores that persisted after limited adjustment for concomitant osteotomy at both 12 months (*β* = 13.1, 95% CI: 3.1–23.0, *p* = 0.013) and 24 months (*β* = 22.2, 95% CI: 11.9–32.5, *p* < 0.001), whereas concomitant osteotomy was not significantly associated with MOCART 2.0 scores at either time point (12 months: *p* = 0.498; 24 months: *p* = 0.695) (Table S3). For clinical outcome scores, membrane type was significantly associated with KOOS Symptoms at 12 months (*β* = 16.2, *p* = 0.046), but was not associated with Lysholm scores or any other KOOS subscales at either time point.

## DISCUSSION

This study demonstrated that both periosteal and collagen membrane‐covered A‐ACI achieved comparable significant clinical improvements at 24 months post‐operatively, with no meaningful between‐group superiority in the primary clinical outcomes or complication rates. While Group C showed advantages in MRI and select histological parameters, these structural evaluations were secondary and exploratory in nature. Although Safranin‐O‐positive cartilage‐like tissue was observed in both groups, neither technique restored the organized zonal architecture of native articular cartilage.

The present study showed significant improvement in both Lysholm scores (Group P: 93, Group C: 94) and KOOS subscales at 24 months compared to preoperative values, with no differences between groups. These results are comparable to a previous comparative study of A‐ACI that reported significant improvements from baseline in both Lysholm and KOOS scores at 24 months post‐operatively, with no differences between periosteal‐ and collagen membrane‐covered procedures [18]. Our findings also align with previous reports of periosteal‐covered A‐ACI, where Lysholm scores significantly improved to 86.4–90.7 points at 1–2 years post‐operatively [1, 50, 51]. While studies comparing first‐ and second‐generation ACI showed no differences at 2 years post‐operatively [13], long‐term follow‐up demonstrated superior outcomes with collagen membrane coverage at 10 years [36]. Given reports of declining clinical outcomes after 2 years in ACI procedures, longer follow‐up of A‐ACI is necessary to evaluate the durability of both covering techniques [2, 4, 5, 17].

MRI findings showed MOCART 2.0 scores in Groups P and C were 60 and 70 at 12 months, and 60 and 83 at 24 months post‐operatively, with Group C demonstrating significant improvement and higher scores particularly in bony defect and subchondral change subscales. Group P scores at 12 months were comparable to previous reports of periosteal‐covered A‐ACI showing scores of 62.5 to 66.6 points at the same time point, but 24‐month scores were lower than prior studies showing 70.4 points at 24 months post‐operatively [1, 50]. This difference might reflect our study's focus on larger defects (≥4.0 cm^2^), as prior A‐ACI studies included smaller lesions with a mean defect size of 3.4 cm^2^ [50]. Larger defect size has been associated with lower MOCART scores after cartilage repair procedures [34], potentially affecting graft maturation and subchondral bone repair. Although the significant imbalance in concomitant osteotomy between the groups may have contributed to the higher MOCART 2.0 scores in Group C, a sensitivity analysis excluding osteotomy cases and multivariable regression analysis adjusting for osteotomy both showed an association between collagen membrane coverage and higher MOCART 2.0 scores that persisted after limited adjustment for osteotomy. These findings raise the possibility that membrane‐related factors may have contributed to the observed MRI differences. The periosteal cambium layer contains osteoblasts and mesenchymal stem cells that secrete inflammatory cytokines acutely and osteogenic factors chronically [23, 45]. These inflammatory and osteogenic factors can promote continuous subchondral bone remodelling, potentially leading to increased bone marrow lesions and increased bone volume despite reduced mineral density [9]. Animal studies have shown subchondral bone sclerosis with periosteal coverage versus normal subchondral bone structure with collagen membrane coverage [45]. Additionally, collagen membranes' superior handling and sealing properties may prevent synovial fluid cytokine infiltration, potentially reducing bone marrow lesions at the graft site [11, 12]. These findings suggest that covering material choice may be related to subchondral bone condition, which is important for the survival and maturation of the cartilage repair tissue.

Our arthroscopic evaluation at a mean of 28 and 26 months post‐operatively in Groups P and C revealed no differences in mean ICRS scores of 9.3 ± 3.4 and 11.3 ± 0.7, respectively, with normal or nearly normal grades achieved in 80% of Group P and 100% of Group C cases. While OAS scores were significantly higher in Group C, this discrepancy is attributable to the difference in scoring criteria: the OAS evaluates graft hypertrophy as one of its five parameters, whereas the ICRS system does not assess this feature [48]. Since graft hypertrophy was observed exclusively in Group P, this scoring difference directly accounts for the discrepancy between the two arthroscopic scores. Consistent with our findings, studies comparing first‐ and second‐generation ACI reported no differences in ICRS scores [13]. In contrast, a previous comparative study of A‐ACI demonstrated significant differences in ICRS scores between groups: 10.7 in the collagen membrane group and 8.3 in the periosteal group [18]. Their periosteal group scores were markedly lower for tibial lesions at 4.3, a lesion location notably absent from our cohort, which likely accounts for our higher periosteal group scores compared to their study. These findings suggest that collagen membrane coverage provides comparable repair tissue appearance to periosteal coverage based on ICRS assessment, while demonstrating better surface characteristics when hypertrophy is considered in the OAS evaluation. However, given the selective nature of second‐look arthroscopy and the small number of cases evaluated, these arthroscopic findings should be interpreted as exploratory trends rather than definitive advantages.

Our histological evaluation demonstrated significantly higher ICRS II scores in Group C for tissue morphology, basal integration, subchondral bone abnormality, and superficial assessment, while overall assessment scores were similar between groups. A previous study of periosteal‐covered A‐ACI reported overall assessment scores of 70.4 points, comparable to our findings, with dense extracellular matrix and Type II collagen formation [1]. Comparative studies between first‐ and second‐generation ACI demonstrated that collagen membrane coverage resulted in superior outcomes in cellular morphology, hyaline cartilage formation, and surface characteristics compared to periosteal coverage [31]. Similar to these studies, we observed better surface characteristics and hyaline cartilage formation with collagen membrane coverage. However, our findings differed in two aspects: we found comparable cellular morphology between groups and notable differences in subchondral bone quality. These contrasting results may be attributed to the unique properties of A‐ACI, which utilizes three‐dimensional culture in atelocollagen rather than suspended chondrocytes used in conventional ACI [39, 40]. Our histological examination confirmed the subchondral bone changes with sclerosis observed in Group P. As discussed earlier, these changes are likely due to the biological properties of the periosteal cambium layer, which secretes inflammatory and osteogenic factors that promote subchondral bone remodelling [23, 45]. The altered subchondral bone environment could influence the overlying cartilage through mechanical and biological interactions, potentially affecting tissue morphology and matrix formation [46]. The Safranin‐O positivity observed in the subchondral bone region in some specimens likely reflects the combined effects of surgical debridement down to the subchondral bone and subsequent biological remodelling of the osteochondral unit. Surgical preparation of the subchondral bone has been shown to allow proteoglycan‐rich repair tissue formation within subchondral voids [19], and remodelling cartilage grafts can exhibit subchondral bone plate changes [3]. Combined with the absence of a clear tidemark in the repair tissue, these factors lead to a blurred boundary where regenerated cartilage‐like tissue extends into the subchondral region. These findings suggest that while both coverage methods achieve comparable overall histological scores, collagen membrane may offer advantages in maintaining better surface characteristics, cartilage tissue quality, and subchondral bone quality. However, the typical organized zonal architecture observed in normal native cartilage was absent in both groups, suggesting that further optimization of ACI techniques may be needed to achieve complete cartilage restoration.

Although the difference did not reach statistical significance (*p* = 0.202), the complication rate tended to be higher in Group P than in Group C (46% vs. 15%). In Group P, complications included graft hypertrophy in three cases and graft delamination in three cases, all requiring arthroscopic debridement. A previous report of A‐ACI with 69 patients and approximately 2‐year follow‐up reported a significantly lower overall complication rate with collagen membrane coverage at 8.6% compared to 29.4% with periosteal coverage, with notably no graft hypertrophy observed in either group [18]. The authors attributed the absence of hypertrophy to careful surgical technique, including uniform periosteal thickness harvesting and appropriate suturing tension, with all surgeries performed by a single experienced surgeon. However, they reported other complications such as hydrarthrosis and knee contracture [18]. In contrast, our study showed higher overall complication rates and the presence of graft hypertrophy in Group P, which is more consistent with typical expectations for periosteal‐covered ACI [22, 37]. These differences suggest distinct complication profiles between studies, potentially reflecting variations in surgical technique, patient selection, or follow‐up methodology. In Group C, two cases (15%) developed enlarging subchondral bone cysts, with both requiring autologous osteochondral transplantation. This complication rate was within the range of the reported clinical prevalence of subchondral bone cysts following ACI, which ranges from 14.7% to 38.8% [11]. Notably, both cysts occurred at anatomical sites corresponding to all‐suture anchor placement, suggesting that localized mechanical stress and micromotion at anchor insertion sites may contribute to cyst formation [16]. This mechanical vulnerability may be further amplified by the differential subchondral bone responses between membrane types, where the absence of periosteal osteogenic influence under collagen membrane coverage could reduce the bone's resistance to mechanical stress at anchor sites [23, 45]. These observations highlight that while collagen membrane effectively reduces graft hypertrophy, careful monitoring of both the graft and subchondral bone remains important. However, the interpretation of complication rates requires caution given the significantly longer follow‐up in Group P (mean: 67.4 months) than in Group C (mean: 36.1 months). In Group P, four of six complications occurred within the first two years, while two cases of graft delamination were identified at 40 and 55 months post‐operatively, illustrating that complications can develop beyond the current observation window of Group C. The current data did not demonstrate a significant difference in complication‐free survival, although the observed events may suggest different early complication patterns between the groups. Longer follow‐up is needed to determine whether late complications, including subchondral bone cyst progression, may emerge in the collagen membrane group.

The significant imbalance in concomitant osteotomy between groups (Group C: 8, Group P: 1; *p* = 0.015) represents an important potential confounding factor in this study. The interaction between realignment osteotomy and cartilage restoration procedures is highly synergistic. Biomechanical unloading may not only protect the cartilage graft from excessive focal stress but may also contribute to structural cartilage regeneration [25]. Consequently, the addition of an osteotomy is generally associated with improved clinical outcomes, enhanced graft survivorship, a reduced reoperation risk, and more favourable cartilage repair quality [6, 7, 10, 26, 30, 32, 49, 52]. The magnitude of this effect is substantial: combined procedures yield significantly greater improvements in multiple validated clinical subscales compared to isolated ACI [10, 52]. Regarding graft survivorship, even in cases of mild varus malalignment (<5°), HTO has been shown to improve graft survival rates by more than 30% (89.5% vs. 58.3%) [6], and a landmark 10‐year outcome study of 210 ACI patients demonstrated 15‐year survivorship of 88% with concurrent HTO versus 66% without (*p* = 0.01) [32]. Concomitant osteotomy also dramatically reduces the reoperation rate, from 68.7% for isolated ACI to 23.9% [7], with an odds ratio for re‐intervention as low as 0.20 [49]; in the patellofemoral compartment, a nationwide database study similarly demonstrated that concomitant tibial tubercle osteotomy reduced the odds of revision from 10.13 for isolated ACI to 1.75 [30]. Furthermore, regarding the quality of cartilage repair, a recent multicenter investigation using the same atelocollagen‐associated ACI demonstrated that corrective osteotomy in patients with varus alignment resulted in significantly better arthroscopic cartilage repair (ICRS grade) compared with maintaining varus alignment without osteotomy, with the corrected varus group achieving repair status comparable with that of the normal alignment group [26]. To address this confounding factor, we performed a sensitivity analysis excluding all patients with concomitant osteotomy and a multivariable regression analysis adjusting for the presence of osteotomy. The sensitivity analysis demonstrated that the collagen membrane group maintained significantly higher MOCART 2.0 scores at both 12 and 24 months even after exclusion of osteotomy cases, while clinical outcomes showed comparable improvement between groups. The multivariable regression analysis demonstrated an association between collagen membrane coverage and higher MOCART 2.0 scores that persisted after limited adjustment at both time points, whereas concomitant osteotomy was not significantly associated with MOCART 2.0 scores. Although the direct effect of biomechanical unloading on specific MRI and histological subscales has not been fully established, it is plausible that improved joint mechanics may also influence these structural outcomes. These adjusted results should be interpreted with caution given the limited sample size. Furthermore, the structural findings, particularly the histological parameters for which sensitivity analysis was not feasible, were evaluated as secondary and exploratory endpoints and should be regarded as hypothesis‐generating. It should also be noted that Group C was on average 10 years older than Group P (43.8 vs. 33.4 years; *p* = 0.062), and advancing age is associated with decreased chondrocyte synthetic capacity and impaired subchondral bone remodelling [24, 44]. This age‐related biological disadvantage in the Group C may have acted in the opposite direction to the osteotomy‐related biomechanical advantage. However, because age imbalance and osteotomy imbalance may exert opposing effects, the independent contribution of membrane type cannot be isolated in the current study.

This study has several limitations. First, this was not a randomized controlled trial. The sequential study design was unavoidable, as the collagen membrane received National Health Insurance approval in Japan only in 2019, precluding prior randomization. Although all procedures were performed by a single experienced surgeon following a standardized protocol, and no substantive changes in surgical technique, perioperative management, or imaging protocol occurred during the study period, subtle temporal effects such as accumulated surgical experience cannot be fully excluded as potential temporal confounding. Second, as discussed above, there was a baseline age imbalance between the groups, representing an unadjusted biological confounding factor. Third, the depth of the debrided defect varied among the cases, which may have influenced the subchondral bone response and the quality of cartilage repair. Fourth, the high frequency of concomitant procedures may have affected the results. Notably, osteotomy was performed significantly more frequently in Group C than in Group P (8 cases vs. 1 case, *p* = 0.015), reflecting the clinical characteristics of each cohort rather than a systematic difference in surgical strategy. This imbalance was closely linked to lesion location, as trochlear lesions were more frequent in Group C (8 vs. 5) and necessitated tibial tubercle osteotomies, while LFC lesions were more frequent in Group P (5 vs. 1). Given that clinical outcomes, complication rates and histological quality may vary by anatomical location [31, 35, 43], this interaction between lesion location and concomitant osteotomy represents an additional potential confounder. Although the sensitivity analysis and multivariable regression analysis suggested that the association between Group C and higher MOCART 2.0 scores persisted after limited adjustment for osteotomy, the limited sample size restricts the robustness of these adjusted analyses, and the histological findings, for which sensitivity analysis was not feasible, should be regarded as hypothesis‐generating. Additionally, the variability in rehabilitation protocols associated with these concomitant procedures represents a further potential confounding factor that cannot be independently isolated from the effect of the procedures themselves. Fifth, our sample size was relatively small, with an achieved power of 77.2%, marginally below the target of 80%, and regional evaluations were not conducted. Importantly, the sample size was determined based on the Lysholm score as the primary clinical outcome, and no a priori power calculation was performed for the secondary structural endpoints (MOCART 2.0 and ICRS II). The statistically significant differences observed in these structural parameters should therefore be regarded as hypothesis‐generating and interpreted as a foundation for future adequately powered randomized controlled trials. Furthermore, second‐look arthroscopy was performed based on clinical indication rather than as a routine protocol, and histological biopsy was obtained in a further subset conditional on additional informed consent. This two‐stage non‐random selection process may have enriched the evaluated subgroups with cases involving clinical events, particularly in the Group P, while patients with asymptomatic successful outcomes may be underrepresented, introducing a significant selection bias. The variable timing of these evaluations was not adjusted for in the statistical analyses due to the limited sample size. Given that graft maturation is a time‐dependent process [38], the wide variability in evaluation timing, particularly in the Group P with a standard deviation of 19 months, may have influenced the arthroscopic and histological findings, introducing a maturation‐related bias. Consequently, the arthroscopic and histological findings should be considered exploratory and interpreted within the context of these biases. Sixth, there was a lack of independent evaluators for some parameters, and the clinical, radiological and arthroscopic assessments were conducted in an unblinded manner, introducing a potential risk of detection bias. Finally, the follow‐up duration differed significantly between the groups (Group P: 67.4 months, Group C: 36.1 months), and the complication comparison should be interpreted as reflecting the early post‐operative period for Group C. Despite these limitations, this study provides the first multimodal assessment of periosteal versus collagen membrane coverage in A‐ACI, offering valuable insights to guide future research and surgical decision‐making in cartilage repair.

In this study, we evaluated the post‐operative outcomes of periosteal‐ and collagen membrane‐covered A‐ACI using clinical scores, arthroscopy, MRI and histological analysis. Both techniques achieved comparable clinical improvements at 2 years post‐operatively, with distinct complication profiles observed between groups. Although an association between collagen membrane coverage and higher MOCART 2.0 scores persisted after limited adjustment for concomitant osteotomy, these radiological findings, alongside the histological and arthroscopic evaluations, should be regarded strictly as secondary, exploratory and hypothesis‐generating. Furthermore, the organized zonal architecture of native articular cartilage was not restored with either technique.

## AUTHOR CONTRIBUTIONS

All authors contributed to the study conception and design. Material preparation, data collection and surgery were performed by Takuma Kaibara, Yuki Ogawa, Masatake Matsuoka, Koji Iwasaki, Tomohiro Onodera, Kazunori Yasuda and Tomonori Yagi. Histological analysis was performed by Yoshitaka Oda and Zen‐ichi Tanei. Statistical analysis was performed by Takuma Kaibara and Daisuke Momma. The first draft of the manuscript was written by Takuma Kaibara, and all authors commented on previous versions of the manuscript. Critical review and supervision were provided by Eiji Kondo, Shinya Tanaka, Kazunori Yasuda and Norimasa Iwasaki. All authors read and approved the final manuscript.

## FUNDING INFORMATION

The authors have nothing to report.

## CONFLICT OF INTEREST STATEMENT

The authors declare no conflicts of interest.

## ETHICS STATEMENT

All procedures performed in studies involving human participants were in accordance with the ethical standards of the institutional and/or national research committee and with the 1964 Helsinki Declaration and its later amendments or comparable ethical standards. Ethical approval for this study was obtained from the Institutional Review Board of Hokkaido University Hospital (No. 020‐0064). Informed consent was obtained from all individual participants included in the study.

## Supporting information

Supporting file 1.

Supporting file 2.

Supporting file 3.

## Data Availability

The data sets generated during and/or analyzed during the current study are available from the corresponding author on reasonable request.
