# Supplementary material for: Multimodal evaluation of periosteal versus collagen membrane coverage in atelocollagen‐associated autologous chondrocyte implantation for large knee cartilage defects
Source: J Exp Orthop. 2026 Sep 25;13(3):e70924. doi: 10.1002/jeo2.70924 (PMC13614688; doi:10.1002/jeo2.70924)
Supplement: Supplementary file 1 [file JEO2-13-e70924-s001.docx]

**Supplementary Table S1.** Patient-Level Data and Outcomes

| Group | Age | Sex | Cause | Lesion Location | Defect Size (cm²) | Concomitant Procedures | F/U (months) | MOCART 2.0 (12M) | MOCART 2.0 (24M) | OAS | ICRS | ICRS II | Complications |
| --- | --- | --- | --- | --- | --- | --- | --- | --- | --- | --- | --- | --- | --- |
| P | 38 | M | Trauma | MFC | 6 | ACLR | 49 | 45 | - | 10 | 12 | 90 | None |
| P | 28 | M | OCD | LFC | 4 | Microfracture | 47 | 45 | 60 | 4 | 2 | - | Delamination |
| P | 41 | M | Trauma | MFC | 5 | AOT | 103 | 80 | 80 | - | - | - | None |
| P | 42 | M | Trauma | LFC | 4 | Meniscal reconstruction | 103 | 60 | 55 | 7 | 11 | 1 | None |
| P | 47 | M | Trauma | MFC | 5 | ACLR | 24 | 55 | 55 | 8 | 12 | 70 | None |
| P | 16 | M | OCD | LFC | 4 | None | 45 | 55 | 60 | 7 | 11 | 50 | None |
| P | 15 | F | Trauma | LFC | 5 | Meniscal resection | 96 | 60 | 55 | 7 | 11 | 80 | Hypertrophy |
| P | 43 | M | Trauma | Trochlea | 10.5 | AOT | 70 | 70 | 70 | 8 | 12 | 90 | Delamination |
| P | 39 | M | Trauma | MFC, Trochlea | 9 | MOWHTO, Meniscal repair | 80 | 50 | 45 | - | - | - | None |
| P | 16 | M | OCD | MFC | 5 | None | 39 | 70 | - | - | - | - | None |
| P | 26 | M | Trauma | Trochlea | 7 | None | 79 | 65 | - | 4 | 5 | 80 | Delamination |
| P | 52 | F | Trauma | Trochlea | 4 | AOT | 72 | 65 | 70 | 7 | 9 | 70 | Hypertrophy |
| P | 40 | M | Trauma | LFC, Trochlea | 4 | None | 69 | 60 | - | 4 | 8 | - | Hypertrophy |
| C | 15 | F | OCD | LFC | 4 | DFO, AOT | 24 | 60 | 75 | 10 | 12 | 90 | None |
| C | 46 | F | Trauma | MFC | 4 | iVHTO, Meniscal resection | 52 | 75 | 90 | 9 | 11 | 80 | None |
| C | 59 | F | Trauma | MFC | 4.5 | MOWHTO, Meniscal repair | 47 | 55 | 85 | 9 | 11 | 85 | Subchondral bone cyst |
| C | 43 | M | Trauma | Trochlea | 8 | AOT | 42 | 70 | 80 | 8 | 12 | 75 | None |
| C | 45 | F | Trauma | Trochlea | 4 | Revision ACLR | 42 | 65 | 80 | 7 | 10 | 75 | None |
| C | 32 | M | Trauma | Trochlea | 4 | PCLR | 33 | 80 | 75 | 9 | 12 | - | None |
| C | 43 | M | Trauma | Trochlea, MFC | 5.25 | None | 43 | 70 | 85 | - | - | - | Subchondral bone cyst |
| C | 53 | F | Trauma | Trochlea | 5 | None | 53 | 75 | - | - | - | - | None |
| C | 43 | M | Trauma | Trochlea | 6 | TTO | 31 | 70 | - | 7 | 11 | - | None |
| C | 41 | F | Trauma | MFC | 4 | iVHTO, TTO | 30 | - | 90 | - | - | - | None |
| C | 54 | M | Trauma | Trochlea | 6.25 | iVHTO, TTO | 24 | 75 | - | 9 | 12 | 70 | None |
| C | 52 | M | Trauma | Trochlea | 5 | iVHTO, TTO | 24 | - | - | - | - | - | None |
| C | 36 | F | Trauma | MFC | 4 | MOWHTO | 24 | 90 | - | - | - | - | None |

Group P, periosteal-covered A-ACI; Group C, collagen membrane-covered A-ACI; OCD, Osteochondritis Dissecans; MFC, Medial Femoral Condyle; LFC, Lateral Femoral Condyle; ACLR, anterior cruciate ligament reconstruction; PCLR, posterior cruciate ligament reconstruction; MOWHTO, Medial Opening Wedge High Tibial Osteotomy; iVHTO, inverted V-shaped High Tibial Osteotomy; DFO, Distal Femoral Osteotomy; TTO, Tibial Tubercle Osteotomy; AOT, Autologous osteochondral transplantation; F/U, Follow Up; OAS, Oswestry Arthroscopy Score; ICRS, International Cartilage Repair Society (arthroscopic score); ICRS II, International Cartilage Repair Society II (histological score). -, indicates data not available or procedure not performed.
