# Supplementary material for: Multimodal evaluation of periosteal versus collagen membrane coverage in atelocollagen‐associated autologous chondrocyte implantation for large knee cartilage defects
Source: J Exp Orthop. 2026 Sep 25;13(3):e70924. doi: 10.1002/jeo2.70924 (PMC13614688; doi:10.1002/jeo2.70924)
Supplement: Supplementary file 2 [file JEO2-13-e70924-s002.docx]

**Supplementary Table S3.** Multivariable Linear Regression: Independent Effects of Membrane Type and Concomitant Osteotomy

| Outcome | Time point | β (Membrane)  [95% CI] | *p*  value | β (Osteotomy)  [95% CI] | *p* value | R² |
| --- | --- | --- | --- | --- | --- | --- |
| Lysholm | 12 | 9.9 [-5.6 to 25.4] | 0.196 | -6.7 [-22.6 to 9.1] | 0.384 | 0.087 |
|  | 24 | 1.4 [-11.5 to 14.4] | 0.817 | 3.5 [-11.1 to 18.1] | 0.624 | 0.026 |
| KOOS |  |  |  |  |  |  |
| Symptoms | 12 | 16.2 [0.3 to 32.0] | 0.046 | -9.0 [-25.1 to 7.2] | 0.260 | 0.204 |
|  | 24 | 1.9 [-11.8 to 15.5] | 0.776 | -3.3 [-18.2 to 11.7] | 0.652 | 0.010 |
| Pain | 12 | 9.2 [-9.3 to 27.7] | 0.312 | -7.4 [-26.3 to 11.6] | 0.427 | 0.054 |
|  | 24 | -3.1 [-14.5 to 8.3] | 0.574 | 0.8 [-11.7 to 13.2] | 0.901 | 0.017 |
| ADL | 12 | 3.5 [-8.2 to 15.2] | 0.535 | -10.2 [-22.2 to 1.7] | 0.089 | 0.176 |
|  | 24 | -4.5 [-13.0 to 4.0] | 0.286 | -2.0 [-11.4 to 7.3] | 0.655 | 0.110 |
| Sport/Rec | 12 | -0.7 [-39.9 to 38.5] | 0.971 | -9.1 [-49.2 to 30.9] | 0.638 | 0.027 |
|  | 24 | -19.1 [-41.0 to 2.8] | 0.084 | -7.1 [-31.2 to 16.9] | 0.543 | 0.238 |
| QOL | 12 | -6.6 [-32.9 to 19.7] | 0.606 | -9.6 [-36.5 to 17.3] | 0.466 | 0.131 |
|  | 24 | -16.4 [-38.1 to 5.4] | 0.132 | 2.5 [-21.3 to 26.4] | 0.826 | 0.122 |
| MOCART 2.0 | 12 | 13.1 [3.1 to 23.0] | 0.013 | -3.6 [-14.5 to 7.3] | 0.498 | 0.279 |
|  | 24 | 22.2 [11.9 to 32.5] | <0.001 | -2.1 [-13.4 to 9.2] | 0.695 | 0.634 |

β, unstandardized regression coefficient; CI, confidence interval; R², coefficient of determination. KOOS, Knee injury and Osteoarthritis Outcome Score; ADL, Activities of Daily Living; Sport/Rec, Sports and Recreation; QOL, Quality of Life; MOCART, Magnetic Resonance Observation of Cartilage Repair Tissue.
