# Supplementary material for: Multimodal evaluation of periosteal versus collagen membrane coverage in atelocollagen‐associated autologous chondrocyte implantation for large knee cartilage defects
Source: J Exp Orthop. 2026 Sep 25;13(3):e70924. doi: 10.1002/jeo2.70924 (PMC13614688; doi:10.1002/jeo2.70924)
Supplement: Supplementary file 3 [file JEO2-13-e70924-s003.docx]

**Supplementary Table S2. Sensitivity Analysis: Between-Group Comparison After Exclusion of Patients with Concomitant Osteotomy (Periosteum, n = 12 vs Collagen Membrane, n = 5)**

| Outcome | Time Point | Group P | Group C | *p* value |
| --- | --- | --- | --- | --- |
| Lysholm | Preoperative | 74.0 (72.8–79.2) | 58.0 (48.0–67.0) | 0.017 |
|  | 12 Months | 82.0 (72.8–96.0) | 92.5 (91.2–93.8) | 0.583 |
|  | 24 Months | 94.0 (79.5–99.0) | 90.0 (81.0–94.0) | 0.634 |
| KOOS |  |  |  |  |
| Symptoms | Preoperative | 67.9 (53.6–81.3) | 32.1 (32.1–71.4) | 0.368 |
|  | 12 Months | 76.8 (64.3–86.6) | 92.9 (91.1–94.6) | 0.170 |
|  | 24 Months | 85.7 (75.0–97.3) | 89.3 (85.7–89.3) | 0.711 |
| Pain | Preoperative | 80.5 (66.0–83.3) | 47.2 (41.7–80.6) | 0.063 |
|  | 12 Months | 90.3 (70.1–97.2) | 88.9 (87.5–90.3) | 0.927 |
|  | 24 Months | 95.8 (86.1–97.9) | 83.3 (80.6–86.1) | 0.152 |
| ADL | Preoperative | 79.4 (70.6–89.3) | 66.2 (61.8–82.4) | 0.205 |
|  | 12 Months | 97.0 (91.9–98.9) | 92.6 (91.2–94.1) | 0.406 |
|  | 24 Months | 97.8 (91.5–100.0) | 85.3 (85.3–94.1) | 0.038 |
| Sports/Rec | Preoperative | 20.0 (8.8–52.5) | 15.0 (15.0–30.0) | 0.915 |
|  | 12 Months | 65.0 (30.0–80.0) | 55.0 (45.0–65.0) | 0.927 |
|  | 24 Months | 77.5 (73.8–96.2) | 60.0 (40.0–70.0) | 0.038 |
| QOL | Preoperative | 31.3 (25.0–39.1) | 18.8 (12.5–18.8) | 0.016 |
|  | 12 Months | 59.4 (48.5–89.1) | 56.2 (53.1–59.4) | 0.783 |
|  | 24 Months | 75.0 (67.2–95.3) | 68.8 (50.0–68.8) | 0.079 |
| MOCART 2.0 | 12 Months | 60.0 (55.0–66.2) | 70.0 (70.0–75.0) | 0.033 |
|  | 24 Months | 60.0 (55.0–70.0) | 80.0 (78.8–81.2) | 0.020 |
| OAS |  | 7.0 (4.8–7.8) | 8.0 (7.5–8.5) | 0.253 |
| ICRS (Arthroscopy) |  | 11.0 (8.2–11.8) | 12.0 (11.0–12.0) | 0.336 |
| ICRS II (Histology) |  | 75.0 (65.0–82.5) | 75.0 (75.0–75.0) | N/A† |

Values are expressed as median (interquartile range). P values were calculated using the Mann–Whitney U test. Available sample sizes at each time point were as follows: clinical outcomes, Group P = 12 and Group C = 5; MOCART 2.0, Group P = 12 and Group C = 5; Arthroscopic evaluation, Group P = 10 and Group C = 3; Histological evaluation, Group P = 8 and Group C = 2. † Statistical comparison was not performed for histological parameters due to insufficient sample size in Group C (n = 2). Group P, periosteal-covered A-ACI; Group C, collagen membrane-covered A-ACI; A-ACI, atelocollagen-associated autologous chondrocyte implantation; KOOS, Knee injury and Osteoarthritis Outcome Score; ADL, Activities of Daily Living; Sport/Rec, Sports and Recreation; QOL, Quality of Life; MOCART, Magnetic Resonance Observation of Cartilage Repair Tissue; OAS, Oswestry Arthroscopy Score; ICRS, International Cartilage Repair Society.
